# Lessons from the stigma of COVID-19 survivors: A Marxist criticism appraisal

**DOI:** 10.3389/fpubh.2023.1156240

**Published:** 2023-03-30

**Authors:** Jin-Long Lin, Yu-Kun Wang

**Affiliations:** ^1^School of Marxism, Tsinghua University, Beijing, China; ^2^School of Sociology and Law, Shanxi Normal University, Taiyuan, China

**Keywords:** COVID-19 survivors, stigma, inequity, social care policy, Marxism

## Abstract

Stigma refers to devalued stereotypes that create barriers for stigmatized individuals. During the COVID-19 pandemic, the stigmatization of survivors worsened existing inequalities and triggered mass hysteria. The paper delves into the stigmatization experienced by COVID-19 survivors and the role of Marxist criticism in analyzing this issue. The main findings from the empiricist tradition approach suggest that the perception of COVID-19 stigma is higher among those who are older, belong to ethnic minorities, lack social support, have manual occupations, and possess lower levels of education. The proposed destigmatization pathways include psychological counseling services, social support, and health education. Employing a Marxist perspective can aid in illuminating how economic practices and material conditions influence prevalent ideologies related to stigma. The stigmatization of COVID-19 survivors may be perceived as a consequence of social power inequality, although the current emphasis on individual characteristics as triggers for stigma may neglect the wider systemic forces in operation. Thus, it’s crucial to establish improved social care policies to combat exploitation and oppression due to power imbalances. The ultimate objective of such an examination is to identify effective approaches to tackle and eradicate stigma regarding health-related concerns. An interdisciplinary approach integrating a pluralistic perspective would benefit investigating how social systems and individual attributes contribute to the exacerbation of social inequality and stigmatization.

## 1. Introduction

Stigma is the epitome of the disharmonious relationship between society and human beings (1, 2). Goffman (3) conceptualized this phenomenon as the “spoiling of identity”; that is, when certain types of people have status standards that do not meet social expectations, they will be demeaned. Disease is one of the most common sources of stigma. During the 2019 coronavirus disease (COVID-19) pandemic, there is no doubt that people infected with COVID-19 were stigmatized around the world (4–9). This stigma has brought large amounts of psychological and physical distress to COVID-19 patients (10, 11).

When infectious diseases with low morbidity and mortality are highly stigmatized, the burden of this stigma can have a negative impact on the overall quality of life in a society (12). The stigmatization of COVID-19 survivors has been captured in many studies around the world. Alchawa et al. (13) found that individuals with some characteristics, such as male manual workers and those with low education levels, had a higher perception of stigmatization when they surveyed 404 COVID-19 survivors from 41 countries. Latha et al. (14) found that among 150 COVID-19 survivors interviewed in Visakhapatnam, Andhra Pradesh, India, 29.3% faced social discrimination. In addition, the stigmatization of COVID-19 survivors has been found in Nepal (15), Ghana (11), Tunisia (16) and other places.

COVID-19 stigmatization can be seen as a social process to exclude potential sources of disease, as people potentially carrying the virus may pose a threat to the normal functioning of society (17). However, although COVID-19 survivors were infected at some point, they fully recovered; from a medical point of view, they do not pose a transmission risk for the spread of the epidemic. On the contrary, because of the immunity caused by viral infection, they should have a lower risk of spreading the virus than normal people who have not been infected with COVID-19, yet they have suffered greater stigma. In response to this paradoxical social phenomenon, this article hopes to analyze the social dynamics underlie the stigmatization of COVID-19 survivors.

For stigmatization to occur, power must be exercised (18). Thus, the power inequality generated by class relations is an effective way to understand the stigmatization of COVID-19 survivors. However, the role of social power and class locations in stigma is frequently overlooked, as power differences are often taken for granted and seem natural, universal and unproblematic. Meanwhile, some social care research views social class as commonly observable personal attributes and material conditions (19). This empiricist tradition of social class research neglects the study of unobservable social mechanisms (20, 21). This view also treats social care as a purely public health issue, ignoring the impact of power inequality on social care. Unlike most studies that conceptualize class as an individual attribute to identify causes and interventions of stigma (10, 11), we aim to reveal that the inequality of social power is an important driving force for stigma processes. To this end, the social care system must formulate a new response.

Whether in the early days of the HIV epidemic or the current COVID-19 pandemic, the issue of stigma is an important challenge that accompanies infectious diseases (14). The present article aims to provide a holistic understanding of the experiences of stigmatization as experienced by survivors of the COVID-19 pandemic and where current social care policies need to be improved. An understanding of these issues will also help us to better cope with the stigmatizations that have occurred in the past and that will occur in future epidemics.

## 2. Methods and materials

### 2.1. Searching strategies and data sources

Articles with the phrases “stigma”/“discrimination”/“stereotype” and “COVID-19 survivors”/“recovered COVID-19 patients”/“Post COVID-19” in the title were obtained from Google Scholar, while articles with the phrases “stigma”/“discrimination”/“stereotype” and “COVID-19 survivors”/“recovered COVID-19 patients”/“Post COVID-19” in the title or abstract were retrieved from PubMed, Elsevier and Web of Science. All titles and abstracts identified in the above electronic databases were screened by 2 authors independently of one another. The full text of each selected article was read one by one to ensure all of them met the research criteria. The literature search period required no setting; the data retrieval period ended on November 30, 2022.

Inclusion criteria: research focused on the stigma (or discrimination, stereotype) of “COVID-19 survivors” (or “recovered COVID-19 patients,” “Post COVID-19”).

Exclusion criteria: studies without a clear source; articles mentioning “stigma”/“discrimination”/“stereotype” and “COVID-19 survivors”/“recovered COVID-19 patients”/“Post COVID-19” that did not address the research object of this work; and repeatedly published research.

### 2.2. Marxist criticism

Although the current study of social care is dominated by the empiricist tradition of social class approach, we seek to introduce an alternative approach, a Marxist analysis. This analytical approach promises to advance the study of social care inequalities and provide an intellectual basis for the social change needed to reduce inequalities. The approach of Marxist analysis, elaborated below, can be summarized by a focus on the relations of economic production through the processes of ownership and class, domination and exploitation.

1. Ownership and class. Any system of production requires the deployment of factors of production, which are commonly classified as land, labor, capital, etc. The way in which these factors of production are deployed forms ownership, or in other words, the social relations of economy. When the power over productive resources is unequally distributed among people, these social relations can be described as class relations (22). As noted by Lenin (23): “Classes are groups of people, one of which can appropriate the labor of another, owing to the different places they occupy in a definite system of social economy.”
2. Domination and exploitation. Class locations—the dominant location and exploited location—can be understood as the social positions occupied by individuals within class relations. Exploitation denotes an unjust social position shaped by an asymmetry of power or the unequal exchange of value between workers and their dominators (employers). According to Marxist theory, the phenomenon of domination and exploitation is a characteristic of all class-based societies, not only capitalism. However, in a capitalist society, the exploited are the proletariat and the exploiters would typically be the bourgeoisie (24).

### 2.3. Appraisal process

In this article, the appraisal process is conducted in three steps: First, we review the literature from four perspectives: the demographic and sociological characteristics of the stigmatized groups, the mechanism underlying the stigmatization of “COVID-19 survivors,” the consequences of stigma and the path to destigmatization. Second, we make a critical appraisal based on Marxist analysis and propose how to understand the phenomenon of stigma from the perspective of Marxism. Finally, we summarize the conclusions of this study.

## 3. Current study on stigmatization of COVID-19 survivors

**Figure 1:**
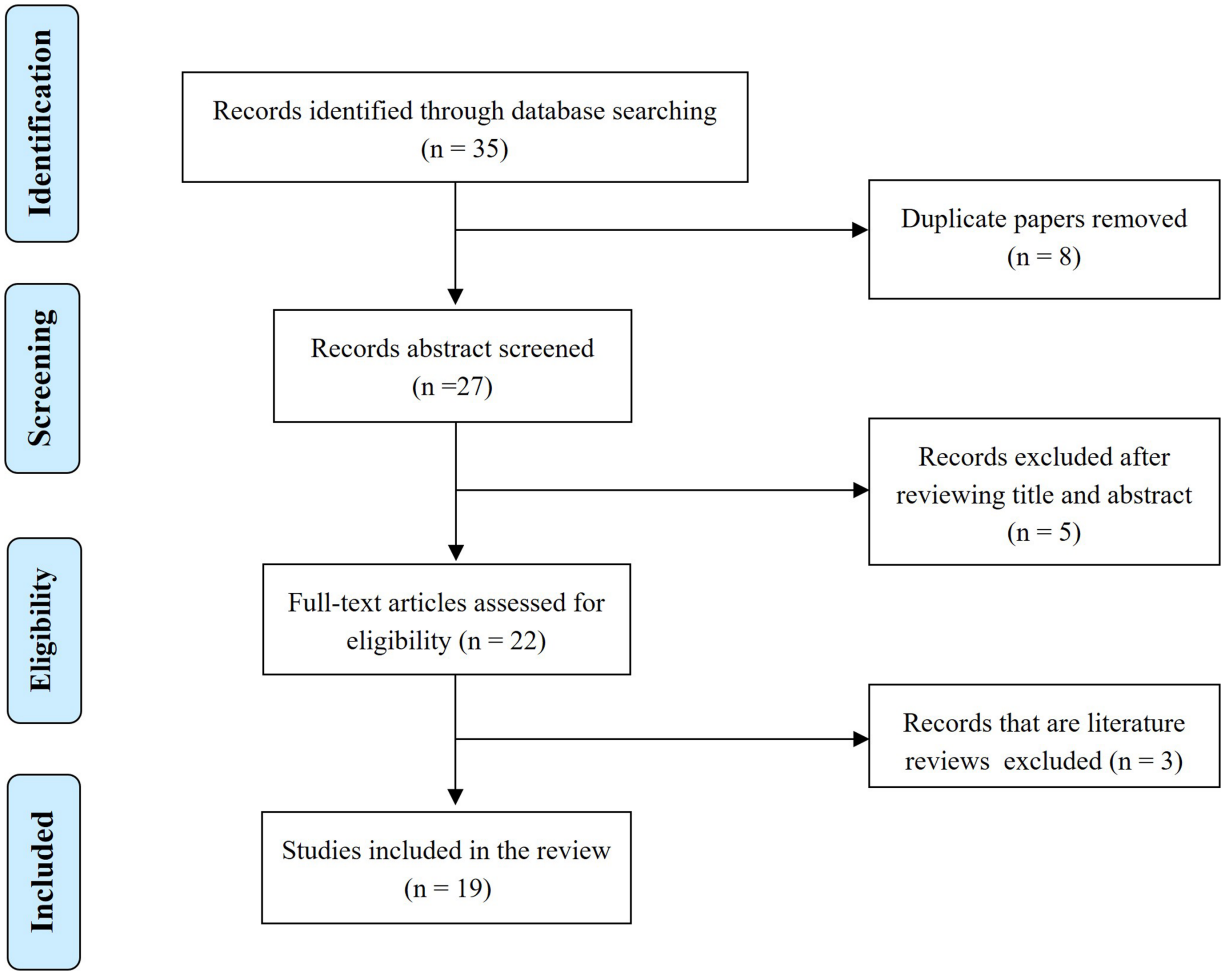
Flowchart of study selection progress.

Although research on the stigmatization of patients with COVID-19 is abundant, the literature on the stigmatization of “COVID-19 survivors” is scarce. The number of articles found in the initial search was 35. The following types of documents were not included: repeated publications, content-irrelevant records and other articles, such as literature reviews (16 articles in total). According to the inclusion criteria and exclusion criteria, the final number of articles that were eligible for this study was 19 (Figure 1). Basic information about the included articles can be found in Table 1.

**Table 1:** Basic information about the included articles.

No.	Study	Study design	Research time	Sample size	Study sites	Main outcome(s)
1	Halouani et al. (16)	Cross sectional	2021.03–2021.05	154	Tunisia	(A) An association between depression and stigma (*p* = 0.002) was found in COVID-19 survivors. (B) Anxiety, depression and post-traumatic stress disorder were independent of the severity of COVID-19.
2	Wahyuhadi et al. (25)	Cross sectional	2020.10–2020.12	547	East Java Province, Indonesia	(A) COVID-19 survivors experienced medium stigma in society and lower quality of life and mental health status. (B) Medium stigma was more likely to be related to quality of life and mental health than low stigma.
3	Fu et al. (26)	Cross sectional	2020.07–2020.09	199	Five cities in China (Wuhan, Shenzhen, Zhuhai, Dongguan and Nanning)	(A) Of all participants, 10.1% reported terrible/poor sleep quality compared to the time before COVID-19. (B) Stigma after recovery was associated with poor sleep quality among COVID-19 survivors, while social support was a protective factor.
4	Amir (6)	Qualitative research	2020.11	30	Kampala, Uganda	(A) COVID-19-related stigma is prevalent, and the most common form of stigma was social rejection and labeling. (B) COVID-19 survivors faced social rejection and community ostracism.
5	Alchawa et al. (13)	Cross sectional	2021.06–2021.08	404	Qatar	(A) More than a quarter of COVID-19 survivors in Qatar had moderate to high perceived stigma. (B) Significant association between perceived stigma and patients’ ethnicity, educational status and type of occupation.
6	Yuan et al. (7)	Cross sectional	2020.05–2020.09	154	ChongQing, China	(A) COVID-19-related stigma is commonly experienced among COVID-19 survivors. (B) Appropriate psychological assistance, public education and anti-stigma campaigns and policies should be enforced to reduce COVID-19-related stigma.
7	Sahoo et al. (9)	Qualitative research	2020.01–2020.05	3	India	Mental health issues, including COVID-19-related stigma, would not have come to the forefront if mental health professionals were not involved in management.
8	Sugiyama et al. (27)	Cross sectional	2020.08–2021.03	140	Hiroshima Prefecture, Japan	(A) Experiences of stigma and discrimination were reported by 43.3% of participants. (B) Significant impacts of long COVID on health in local communities.
9	Yadav et al. (28)	Cross sectional	2020.10–2020.11	122	Delhi, India	(A) Statistically significant association between feeling ashamed and blaming themselves for COVID-19 (*p* = 0.046). (B) The stigma related to COVID-19 needs to be tackled with a multipronged strategy.
10	Atinga et al. (29)	Qualitative research	2020.03–2021.02	45	Greater Accra Region, Ghana	Everyday lived experiences of the participants were disrupted with acts of indirect stigmatization, direct stigmatization, barriers to realizing a full social life and discriminatory behaviors across socioecological structures.
11	Campo-Arias et al. (30)	Cross sectional	2020.10–2021.04	330	Santa Marta, Colombia	(A) Depression, insomnia and post-traumatic stress were significantly associated with the discrimination perceived by COVID-19 survivors. (B) Perceived discrimination is a social stressor that affects the psychological well-being of people recovered from COVID-19.
12	Damant et al. (31)	Longitudinal Cohort	2021.05–2022.05	145	Canadian City of Edmonton, Alberta	(A) Total stigma score was positively correlated with symptoms, depression, anxiety, loneliness, reduced self-esteem, thoughts of self-harm, post-COVID functional status, frailty, EQ5D5L score and number of ED visits. (B) Total stigma score was negatively correlated with perceived social support, 6-min walk distance and EQ5D5L global rating.
13	Osei et al. (32)	Cross sectional	2020.10–2020.11	3,529	Ghana	Because of the relatively high proportion of poor knowledge and negative attitudes toward COVID-19, stigma and discriminatory tendencies were consequently high.
14	Iqbal et al. (33)	Cross sectional	2020.09–2020.12	158	Karachi, Pakistan	Long-COVID syndrome, including COVID-19-related stigma, is similar to the post-discharge manifestations of the survivors of prior pandemics (SARS and MERS).
15	Winugroho et al. (34)	Cross sectional	2020.12–2021.03	63	Central Java Province, Indonesia	Stigma is an important predictor that affects mental toughness and quality of life to increase immunity for nurses themselves.
16	Siregar and Purba (35)	Experiment research	–	25	Indonesia	There was no significant reduction in stigma after intergroup contact, for example, a video conference.
17	Adhikari et al. (8)	Cross sectional	2020.09–2021.01	303	18 districts located in 7 States in India	(A) Of the COVID-19 survivor participants, 38.6% reported experiencing severe stigma. (B) Study recommends the timely dissemination of accurate information to populations vulnerable to misinformation and psychosocial interventions for individuals affected by stigma.
18	Latha et al. (14)	Cross sectional	2020.10–2020.11	150	Visakhapatnam, India	Social discrimination among participants was greater with increased age, female gender, among educated people, in joint families, among married individuals, in upper social class and for those who had a long hospital stay; however, it was not significantly associated statistically.
19	Dar et al. (36)	Cross sectional	2020.04–2020.06	91	Kashmir, India	(A) High levels of enacted and externalized stigma among COVID-19 survivors. (B) Enacted stigma was greater among males and in those who were highly educated.

### 3.1. Characteristics correlated with stigma among COVID-19 survivors

Many studies have shown the pervasiveness of the stigmatization of COVID-19 survivors. For example, in Qatar, more than a quarter of COVID-19 survivors reported moderate to high levels of stigma (13). In another study, discrimination in Hiroshima Prefecture, Japan, among COVID-19 survivor participants reached 43.3% (*N* = 140) (27).

Current studies on the perception of stigma in COVID-19 survivors have found the following characteristics to correlate with stigma: (1) Age: Older COVID-19 survivors (≥60 years) experience higher social discrimination than COVID-19 survivors in other age groups (14). (2) Ethnicity: Ethnicity is one of the factors affecting the perceived stigma of COVID-19 survivors, with non-Arabs having a much higher perception of stigma than Arabs in Qatar (13). (3) Social support: Social support is a powerful weapon against stigma; thus, groups such as solitary populations and migrant workers have a higher risk of stigma and higher levels of COVID-19-related stigma perception (13). (4) Occupation: It has been shown that manual workers have higher levels of perceived stigma than those practicing professional occupations (13). However, health-care workers are a special case: COVID-19 survivor nurses have reportedly endured a higher degree of stigma (34). (5) Education level: Lower levels of education may result in higher perceived COVID-19 stigma (36). The more people lack understanding about how COVID-19 is spread, the more likely they are to experience COVID-19 stigma (14). Therefore, education can help prevent stigma by increasing awareness about the disease and reducing the likelihood of experiencing COVID-19 stigma (13).

### 3.2. Mechanisms causing stigma

It has been shown that the stigmatization of COVID-19 survivors stems from two main sources, namely enacted stigma and internalized stigma.

On the one hand, as far as enacted stigma is concerned, it mainly originates from the community. Recovered COVID-19 patients often suffer from stigmatization and discrimination, and their communities have an irrational fear of them (33). In a study involving 199 COVID-19 survivors, 30% of the participants confessed to having experienced rejection by their neighbors or community (26). Alchawa et al. (13) found that some workers in specific occupations that require contact with many people, such as grocery store clerks and delivery drivers, were more at risk for experiencing stigmatization; this is because they were accused of bringing disease into the community. There are also communities where elders pray for divine intervention and spiritual protection to help the community in the fight against COVID-19 when they believe that human power alone can no longer stop the spread of the virus; however, this can also increase stigma because once someone is infected with COVID-19, he is treated as an offender to the gods (29).

On the other hand, internalized stigma is also a source of stigmatization. In the context of a global outbreak of COVID-19, the fear of the virus devalues the social status of those associated with it. This phenomenon creates negative self-image of the prejudiced group, which triggers their internalized self-stigma. In Santa Marta, Colombia, 32.12% of COVID-19 survivors (*N* = 330) had high perceptions of stigma, which increased their risk of depression and insomnia (30). Almost all existing studies point out that stigma originates from fear. Suryandari (37) argued that the reason for COVID-19 stigma is because COVID-19 is a new disorder, and people become afraid of associating with other people because of their fear of the unknown. Due to the lack of knowledge about the new virus, people may misunderstand the infection, and some may even see it as a curse or sin (14).

### 3.3. The psychosocial impact of COVID-19 stigma on survivors and society

The COVID-19 pandemic triggers widespread stigma toward those who have contracted the virus or are associated with it, causing psychosocial problems and potential societal outbreaks. A number of studies have shown that stigma can put COVID-19 survivors under greater stress, which, in turn, can lead to many negative social consequences (16, 25, 31). It can be seen in various ways: (1) Once a generalized COVID-19-related stigma develops, it can lead to serious psychosocial problems and mental instability (29). High levels of perceived stigma can cause depression and insomnia in COVID-19 survivors (30). (2) Stigma is not only directed at COVID-19 survivors, but also at their family members, which can lead to self-blame and further jeopardize their psychological well-being (14). (3) Severe stigma may encourage people to avoid stigmatization by hiding the disease. This, in turn, will likely lead to a social outbreak of the virus (37). (4) In the long run, this may lead to social catastrophe. Stigmatization can undermine social cohesion and lead to the social ostracism and isolation of COVID-19 survivors (37).

### 3.4. Suggested responses to counter stigma

To support COVID-19 survivors in overcoming this crisis and prevent the emergence of larger social problems, existing research primarily proposes destigmatization pathways such as psychological counseling services, social support, and health education.

1. Psychological counseling services: Professional psychological help can be an effective response to mental health problems. From the beginning of discharge preparation, we should carry out actions to reduce the intrinsic stigma of COVID-19 survivors (30), where early psychological interventions can reduce the long-term adverse effects of mental illness due to stigma (38).
2. Social support: COVID-19 survivors are vulnerable when returning to their communities, as they face stigmatization (25). Therefore, the dissemination of this information in the community should be discouraged and community support should be provided (39). If COVID-19 survivors and their families are at risk of physical attacks in the community, policies and regulations to protect them should also be developed and improved (40). But simply encouraging intergroup contact and communication seems less effective in reducing stigma (35).
3. Health education: In the face of stigma, it is necessary to pay attention to social health education work. In the information age, appropriate and reliable ways to disseminate COVID-19-related information should be promoted (32). It is important to dispel the idea that COVID-19 survivors are still contagious after recovery (32, 33). Health education regarding COVID-19 can be carried out through various authoritative media channels such as public service announcements, newspapers and television programs.

## 4. Marxist criticism on stigmatization of COVID-19 survivors

Most current studies conceptualize the mechanisms underlying the stigmatization of COVID-19 survivors as a set of attributes of the individual, while the power inequality generated by class relations is somewhat neglected. Thus, Marxist analysis is introduced in this paper to reveal additional knowledge on social care policies learned from the stigmatization of COVID-19 survivors.

### 4.1. The complex nature of stigmatization beyond individual attributes

Stigmatization is a form of violence committed by people who tend to stigmatize against certain groups. Existing studies have identified some characteristics of stigmatized groups, such as manual workers and those with a low education level (13). However, studies on individuals with stigmatizing tendencies have not found specific characteristics. For instance, in a study by Osei et al. (32), some socio-demographic characteristics such as education, marital status, employment and religion did not significantly predict the propensity to stigmatize COVID-19 survivors. This indicates that explaining stigma solely in terms of individual sociodemographic characteristics is not sufficient.

Although existing studies summarize the characteristics of stigmatized groups, these individual characteristics are only superficial features. Many risk factors, such as lower social support, lower-paid occupations, and lower levels of education, are commonly concentrated in certain groups. Therefore, the issue of stigmatization cannot be exclusively explained by individual factors. Instead, it is embedded in fundamental political, socioeconomic, and philosophical problems. To understand the mechanisms underlying stigmatization, it is necessary to introduce the perspective of social power and social relations. Marxism provides a framework that can effectively address this requirement. According to Marxist theory, public health and its related issues are products of capitalist domination and the reproduction of dominant class ideology (41, 42). Similarly, COVID-19-related stigma is not solely a medical issue; it is also about ideology and capital logic.

### 4.2. The influence of capitalist systems on the stigmatization of COVID-19 survivors

By applying Marxist criticism to the stigmatization of COVID-19 survivors, valuable insights can be gained into how this phenomenon is deeply entrenched in larger structures of power and exploitation. Specifically, Marxist analysis reveals how capitalist systems have played a significant role in perpetuating and exacerbating the stigmatization of COVID-19 survivors.

Firstly, capitalism increases the risk of spreading infectious diseases and stigma. Capitalism spreads the disease by impoverishing migrant health and blaming them, where territorial, political, judicial and economic expulsions are their means (43). Siu’s study (44) of social and cultural values found that the vulnerability of some groups to stigma is reinforced under the capitalist ideology. It has also been shown that workers with the least power and resources are overlooked because they do not have easy access to infectious disease-related testing, and they are more often stigmatized (45). It is no wonder that Henderson (46) argued, “surely it is time the medical profession objected publicly and loudly to being manipulated by government and the corporate interests it transparently serves.”

Secondly, capitalism has kidnapped science, which can no longer be truly objective or independent. As criticized by McClure et al. (47), in the context of COVID-19, epidemiology now focuses the obtention of viral infections on individual biology and behavior. However, this ignores the influential role of the occupational environment in the transmission of the virus and, to some extent, absolves industry of responsibility for worker safety. In addition, there are studies showing that public and political attitudes toward masks need to rely more on scientific evidence. Such evidence, in addition to including epidemiological and infectiological information, should also consider its social and personal significance; otherwise, it may harm the interests of marginalized groups (48, 49).

Thirdly, capitalists have used the pandemic in their interest, resulting in workers facing harsher living conditions and a higher risk of infection, which exacerbates their stigma. As noted by Link and Phelan (50), “stigma power” is a resource that exploits and suppresses others through stigma. Although this process may manifest itself in all aspects of society, it is more visible and easier to capture in the workplace. For example, one study from Visakhapatnam confirmed this phenomenon. Wage cuts, company firings for being deemed unproductive and more have been observed with some COVID-19 survivors (14). In addition, when it comes to hiring employees, some companies even see the economic turmoil as an opportunity to hire workers on unsafe contracts (51).

### 4.3. The vicious circle of stigma and social inequality

While existing studies focus mainly on the psychosocial impact of COVID-19 stigma on survivors and society, little attention has been paid to how this stigmatization reinforces capitalist inequality mechanisms (29, 31). A discussion of this topic is crucial, as the mechanism of capitalist inequality has a direct impact on the allocation of public health resources, the improvement of the social care system, and the health and dignity of economically and socially disadvantaged groups who are more susceptible to stigmatization during epidemics.

The reproduction of stigma and social inequality reinforces each other, particularly for marginalized groups that are often hardest hit by stigma due to weak health, poverty, and low education levels (13). Stigma becomes a separate force and resource for control, subjugation, and exploitation in the hands of power by creating a division between stigmatized and non-stigmatized individuals, reinforcing existing power structures and maintaining the status quo. Stigmatized individuals may be excluded from opportunities and kept in a state of subjugation and dependence, while power holders use pandemic fear to justify increased control and restrictions, further reinforcing their control. This vicious circle leads to the reproduction of poverty, deteriorating health, and social inequality, increasing the risk of stigmatization (14, 51). Thus, it seems that this vicious circle is one of the social mechanisms that create stigma. In conclusion, “market incentives in capitalist economies and public health requirements are contradictory” (41).

### 4.4. A Marxist approach to social care policy

The social care system is a critical component of our social infrastructure, and the pandemic has highlighted the cost of neglecting it. Presently, social care policies that address stigma mostly focus on the healthcare sector, such as promoting public health knowledge, strengthening the psychological resilience of COVID-19 survivors, and correcting attitudes toward the virus (14, 32, 40). However, research has suggested that social support is necessary to eradicate stigma, but it has not yet revealed the social mechanisms of stigma or how to eliminate it from the perspective of social power inequality (25, 39).

Stigmatizing COVID-19 survivors is not just a problem of health information asymmetry and fear caused by ignorance. Dealing with this stigma requires more than avoiding the disclosure of private information or using the correct terminology (26, 28, 29). It is crucial to recognize that the health field is not the only component of the social care system. Capital’s profit motive may undermine healthcare systems by misallocating medical resources (46). Therefore, it is essential to establish better social care policies to counteract exploitation and oppression under power inequalities. If social care policies do not address the root causes of COVID-19-related stigma, such as the social determinants of marginalized people facing economic instability, they will continue to suffer from stigmatization, especially during times of crisis (52).

Firstly, Respect for all jobs, including low-skill and low-wage jobs, is crucial. These jobs often involve manual labor that requires contact with many people and provides limited social support and avenues of vocalization. Therefore, those in these jobs who become infected with COVID-19 are vulnerable to stigmatization (13), as they are allocated fewer social resources in the existing system. As a result, they may face more severe mental health problems and stress. However, the division of labor contributes to societal efficiency and low-skill jobs play an important role in society, particularly during an epidemic. As noted by International Labor Organization (ILO) (53), the COVID-19 pandemic may facilitate the erosion of the high skill/low skill distinction and encourage a re-evaluation of the socio-economic worth of certain occupations.

Secondly, Strengthening the power of trade unions across various industries can contribute to social care, especially in light of the deep-seated inequalities revealed by the COVID-19 crisis, particularly in the social care sector. From a trade union perspective, investing in workers in healthcare and informal sectors is crucial. Studies have shown that informal healthcare and migrant workers are stigmatized and at-risk groups, facing low formalization, wages, and unstable work hours (13, 54). These marginalized groups face economic instability, and given their tendency to minimize self-expression and avoid disclosing their psychological problems (55), their stigmatization problems are likely underestimated. They require union protection. Thus, highlighted, Workers organizations should regard Covid-19 as a wake-up call, a wake-up call for contributing to building forward better together; and the achievements during the crisis should serve as a steppingstone for a recovery for all, including workers in the informal economy (53).

Thirdly, we should aim to improve the social care system through tax system reform. Despite the fact that the average worker has experienced the longest pay squeeze in living memory over the past decade, total wealth has been growing in an unequal manner (56). Therefore, capitalists or the wealthy should pay their fair share to fix our social care system. In the healthcare industry, for example, studies have shown that with increased social support, the resilience and stress tolerance of healthcare workers can increase (34). We might start by raising the pay of healthcare workers and reforming the tax system and then gradually expand the reforms to create a better social care system.

## 5. Discussion

### 5.1. Main findings from Marxist criticism appraisal on overall literature review

The current study on the stigmatization of COVID-19 survivors has yielded the following key insights. Older age, ethnicity, lack of social support, manual occupation, and lower education levels are associated with higher levels of COVID-19 stigma perception. COVID-19 stigma is mainly thought to stem from enacted stigma (coming from the community) and internalized stigma (negative self-image of the prejudiced group triggered by the devaluation of social status). Considering that stigma can put COVID-19 survivors under greater stress, leading to negative social consequences such as isolation, avoidance, discrimination, and potential societal outbreaks, prompt responses are suggested to counter the stigma, such as psychological counseling services, social support, and health education.

Nonetheless, the discrimination against COVID-19 survivors cannot be solely explained by individual factors. Instead, it is rooted in underlying political, economic, and philosophical issues. In this paper, Marxist criticism is concerned with power dynamics and how they shape the relationships between individuals and groups, during the stigmatization of COVID-19 survivors. Additionally, we look at how the stigmatization of COVID-19 survivors is linked to broader issues of inequality and exploitation. By examining the economic, social, and political structures that underlie this phenomenon, we can identify the root causes of stigmatization and work toward creating a more just and equitable society.

Overall, capitalist systems have played a significant role in the stigmatization of COVID-19 survivors. Their emphasis on individualism, fear, and profit has contributed to a culture of blame and stigma, with COVID-19 survivors being labeled as irresponsible, a threat to economic stability, or even morally deficient. As humans will always coexist with viruses and continue to navigate past and future pandemics, it is essential to recognize and address the ways in which capitalist systems can perpetuate and exacerbate social inequalities and stigmatization. Stigma reinforces the mechanisms of capitalist inequality, particularly for marginalized groups who are often the most adversely affected by discrimination due to their poor health, poverty, and limited education levels. This perpetuates a destructive cycle of stigma and social inequality that directly affects public health resources, the enhancement of social welfare systems, and the well-being and dignity of economically and socially underprivileged communities.

### 5.2. Advantages and limitations of Marxist criticism

Marxist criticism is an analytical approach that can explain how economic structures, power relations, and political forces contribute to the stigmatization of certain groups. However, it is essential to recognize the limitations of the Marxist perspective when it comes to understanding COVID-19-related stigma fully.

On the one hand, Marxist criticism offers significant analytical advantages. First, it enables the elucidation of concerning trends in public health, such as privatized health economies. The power of the upper class and the political economy determinants of social care. This allows for a better investigation and interpretation of the mechanisms underlying COVID-19-related stigma. Second, Marxist criticism believes in our capacity for change and defends indispensable social values, such as creating an equitable society by ending exploitation. Finally, Marxist criticism is a call for engagement to protect these values by deepening opportunities for public participation in shaping collective choices.

On the other hand, it is clear from the above analysis that the Marxist perspective is not a panacea. Although it can explain part of the social mechanisms that shape stigma, there are also aspects that it cannot respond to. For example, in Latha et al.’s study (14), it was found that older people were more likely to be stigmatized than other age groups. In fact, age should indeed be considered as an independent, micro-level predictor of having a risk of being stigmatized. Older individuals are more likely to be stigmatized, and age is associated with factors such as poor physical fitness and a weak immune system. Although Marxists believe that attitudes to old age are influenced by capitalism, they cannot deny that aging is an independent risk factor for health at the medical level (57). In this case, Marxism cannot completely replace the perspective of individual attribute analysis.

### 5.3. Research outlook on lessons from the stigma of COVID-19 survivors

To gain a more objective and comprehensive understanding of stigmatization, an integrated and pluralistic perspective is necessary. Instead of portraying stigmatized groups as limited in terms of individual attributes, the Marxist analytical perspective can enrich the study and explore the social mechanisms of stigma formation from a more macroscopic view. By doing so, researchers can contribute to the establishment of a comprehensive, scientific, and dimensional social care system.

Future research can significantly advance the study of the stigma of COVID-19 survivors by integrating the perspective of Marxism. Specifically, researchers could investigate how capitalist systems perpetuate and exacerbate social inequalities and stigmatization, and how these systems impact the distribution of resources and the well-being of marginalized communities. Additionally, researchers could explore the intersectionality of the stigmatization of COVID-19 survivors with other forms of oppression, such as racism, ableism, and homophobia. By adopting an intersectional approach, researchers can identify the unique challenges that certain groups face and develop targeted interventions to address these issues. Finally, future research can also explore the potential for collective action and social movements to challenge the stigmatization of COVID-19 survivors. Marxist theory emphasizes the importance of collective action and solidarity in challenging power structures and promoting social change. Therefore, research can examine how social movements can challenge the stigmatization of COVID-19 survivors and how this relates to broader struggles for social justice.

## 6. Conclusion

The stigmatization of COVID-19 survivors results from social power inequality, yet current research focuses on individual attributes as the mechanisms of stigma. A Marxist analysis can help expose how material conditions and economic practices shape the dominant ideologies surrounding stigmatization. The goal of this critical appraisal is to identify ways to end the stigma surrounding health-related issues. Current studies limit the contributors to social care to public health policymakers, medical departments, nursing homes, and communities, neglecting the roles and responsibilities of other subjects in social care. From a Marxist class analysis perspective, the responsible subject of social care should not be limited to the traditional subject. The function of trade unions and tax system reform in fixing our social care system should also be taken into consideration. Future research can advance our understanding of COVID-19 survivor stigma and social care reform by highlighting systemic factors that contribute to stigma and identifying avenues for collective action and change. Overall, the creation of a social care policy system is complex, impacted by numerous social factors, and should not only be studied in the field of public health. An interdisciplinary approach will be beneficial in future efforts to build the social care policy system.

## Author contributions

J-LL and Y-KW worked together to conceptualize the research questions and prepare the research protocol, drafted the manuscript, and reviewed and edited the manuscript. All authors contributed to the article and approved the submitted version.

## Conflict of interest

The authors declare that the research was conducted in the absence of any commercial or financial relationships that could be construed as a potential conflict of interest.

## Publisher’s note

All claims expressed in this article are solely those of the authors and do not necessarily represent those of their affiliated organizations, or those of the publisher, the editors and the reviewers. Any product that may be evaluated in this article, or claim that may be made by its manufacturer, is not guaranteed or endorsed by the publisher.
